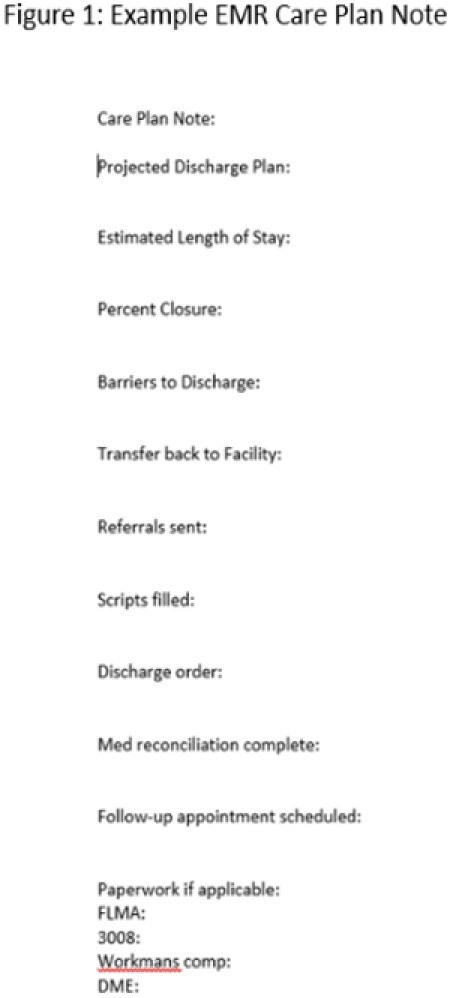# 586 Show Me the Wound Closure! Burn Registry Data Compilation Project

**DOI:** 10.1093/jbcr/irae036.220

**Published:** 2024-04-17

**Authors:** Pamela Michelli, Sandra Martinez, Jeana Swain, Sherrina Richards, Brooke Bozoian

**Affiliations:** Orlando Health Regional Medical Center, Windermere, FL; Orlando Health Regional Medical Center, Orlando, FL; Orlando Health Regional Medical Center, Windermere, FL; Orlando Health Regional Medical Center, Orlando, FL; Orlando Health Regional Medical Center, Windermere, FL; Orlando Health Regional Medical Center, Orlando, FL; Orlando Health Regional Medical Center, Windermere, FL; Orlando Health Regional Medical Center, Orlando, FL; Orlando Health Regional Medical Center, Windermere, FL; Orlando Health Regional Medical Center, Orlando, FL

## Abstract

**Introduction:**

The ABA requires that a verified burn center report wound closure percentage on discharge for patients with burns >10% TBSA. Multiple data elements are entered by the burn registry staff into the registry. One data point, wound closure percentage (WCP) was not being captured consistently in the registry, because the data point wasn’t being reported. The initial rates of wound closure capture for 2021 and 2022 were 7% and 43% respectively and the ABA BCQP platform requires 100% capture of this data element. The purpose of this project is to develop a reliable process for the burn registrars to capture this data element.

**Methods:**

We performed a retrospective review for this quality improvement project using data collected from our burn registry. Inclusion criteria included: burn patients 18 years or older who were admitted to a single burn center that had a TBSA ≥ 10% from January 1, 2021 – August 31, 2023.

Data collected was presented in our multidisciplinary Burn Quality Meeting to optimize ways to capture this data point. Suggested implementations included: Discharge summary documentation in the Electronic Medical Record (EMR), reporting WCP at weekly burn multidisciplinary rounds (MDR). Education was developed and disseminated to physicians and registry staff.

**Results:**

815 burn admissions from January 1,2021 to August 31,2023 were reviewed, 171 patients were admitted with a burn of TBSA ≥ 10%. Discharge summary documentation of WCP was implemented October 2021 and a yearly review demonstrated a 39% capture rate. MDR reporting was implemented November 2022, a seven-month review demonstrated a 58% capture rate.

**Conclusions:**

The initial implementation, discharge summary documentation resulted in very little improvement in capture of WCP. The MDR improved our capture rate further but did not meet the 100% data compliance goal. A daily discharge EMR care plan note was developed to help social work with planning for discharge for our patients to decrease the length of stay rate. It was decided to include WCP in this note to increase the capture rate. Moving forward, we found this to be an opportunity for improvement and will utilize the care plan note in combination with MDR reporting for WCP capture. The combination of MDR reporting and review of the daily discharge EMR care plan note is proving to be the most efficient way to capture WCP for our registry staff. The Burn Clinical Coordinator (BCC) is present for MDR reporting and can enter WCP in the registry as needed.

**Applicability of Research to Practice:**

Utilization of documentation of WCP in the discharge summary, MDR reporting of WCP, and the review of the daily discharge EMR care plan note will allow for 100% compliance of data capture of WCP.